# Methicillin-Resistant *Staphylococcus aureus*, Geneva, Switzerland, 1993–2005

**DOI:** 10.3201/eid1402.070229

**Published:** 2008-02

**Authors:** Patrice Francois, Stephan Harbarth, Antoine Huyghe, Gesuele Renzi, Manuela Bento, Alain Gervaix, Didier Pittet, Jacques Schrenzel

**Affiliations:** * University of Geneva Hospitals, Geneva, Switzerland; 1 These authors contributed equally to this article.

**Keywords:** Methicillin-resistant, Staphylococcus aureus, non-multiresistant, molecular characterization, SCCmec, Switzerland, clonality, epidemiology, dispatch

## Abstract

Molecular characterization of methicillin-resistant *Staphylococcus aureus* (MRSA) strains different from those of an endemic healthcare-associated clone was conducted over 13 years in Geneva, Switzerland. We demonstrated strain diversity, including clones rarely found in Europe. Local epidemiology of community-associated MRSA is diverse and is evolving by importation and transmission of new strains.

Community-associated methicillin-resistant *Staphylococcus aureus* (CA-MRSA) is responsible for severe infections related to carriage of exotoxins such as the Panton-Valentine leukocidin (PVL), toxic shock syndrome toxin 1 (TSST-1), or exfoliatin A (*1*). Genetic content of CA-MRSA strains depends on the local epidemiology and was recently described as polyclonal (*2*) Another study reported more uniformity in CA-MRSA lineages (*3*). We have recently evaluated prevalence of MRSA at hospital admission and showed a low CA-MRSA prevalence, a reservoir of asymptomatic carriers, and a high degree of CA-MRSA diversity (*2*). However, despite an increasing number of CA-MRSA infections in Europe (*4*), few, if any, studies have assessed long-term epidemiology of CA-MRSA in a geographically confined area. Awareness of secular trends in molecular features of CA-MRSA could affect public health and patient care. Therefore, our aim was to evaluate genetic diversity and spread of non–multidrug-resistant MRSA strains isolated in Geneva over a 13-year period.

## The Study

We selected 2 collections of strains with 151 nonduplicated MRSA isolates identified in patients or carriers treated at our institution. The first collection was from a retrospective review of laboratory records and included non–multidrug-resistant (gentamicin- and ciprofloxacin-susceptible) strains collected during 1993–2002 that had a phenotype different from the endemic healthcare-associated MRSA (HA-MRSA) strain in Geneva. The prevalent HA-MRSA clone in Geneva is sequence type (ST) 228-MRSA-I (CC5), which shows resistance to gentamicin, ciprofloxacin, clindamycin, and erythromycin. HA-MRSA strain ST8-MRSA-IV has been sporadically introduced from France. This strain has the same phenotype as ST228-MRSA-I (CC5) except for its susceptibility to gentamicin (*5*). We included all strains resistant to or with intermediate susceptibility to fusidic acid, a characteristic of many CA-MRSA isolates in Europe.

The second collection was isolates selected from patients prospectively identified as colonized or infected with CA-MRSA by the CA-MRSA surveillance program during 2003–2005 (*6*). CA-MRSA was defined as any isolate with an antimicrobial drug resistance profile different from the strain endemic in the Geneva healthcare setting and diagnosed in a patient without a history of hospitalization in the previous 12 months.

MRSA identification was performed by using standard methods (*7*) according to Clinical and Laboratory Standard Institute recommendations (*8*) and confirmed by quantitative PCR (*9*). Genomic DNA isolated from 1 colony was tested by quantitative PCR for staphylococcal cassette chromosome *mec* (SCC*mec*) elements, accessory gene regulator group, and the PVL gene (*10**,**11*). Presence of type V cassette, TSST-1, and exfoliatin toxins was assessed by using specific oligonucleotides (sequences are available at www.genomic.ch/sup6.php). Multiple-locus variable-number tandem repeat analysis, which consisted of a multiplex PCR with 10 primer pairs, and multilocus sequence typing were performed as reported (*11**,**12*).

Since late 2002, all patient demographic and epidemiologic data have been reviewed and recorded on a standardized form by a public health nurse (*6*). For this analysis, we included only those patients who were seen at our institution or outpatient clinic.

A total of 92 strains from 51 patients (55% male, mean ± SD age 37 ± 28 years) were obtained from the retrospective specimen collection. Fifty-nine isolates were obtained from clinical specimens and 33 from screening swabs. Among these isolates, 46 were obtained from skin and soft tissue samples and 13 from other body sites. A total of 59 isolates were obtained from the prospective CA-MRSA surveillance system from 59 patients (mean ± SD age 33 ± 21 years, male:female ratio 2.7).

Table 1 shows that most CA-MRSA strains isolated during 2002–2005 were associated with skin and soft tissue infections. Most cases of infection or colonization were associated with migration or travel history. Four healthcare workers acquired CA-MRSA strains epidemiologically unrelated to each other. In 2 instances, family members of these workers were also affected.

**Table 1:** Demographic characteristics, types of infection, and epidemiologic profiles of  61 patients with CA-MRSA colonization or infection, Geneva University Hospitals, 2002–2005*

Characteristic	Value
Demographic data	
Mean ± SD age, y	33 ± 21
Male	35 (57)
Immigrant, foreign origin or residency outside Switzerland	25 (41)
Recent history of travel before CA-MRSA isolation	29 (48)
Institutionalized (prison, nursing home, asylum-seeker camp)	11 (18)
Healthcare worker	4 (7)
Type of infection/colonization	
Primary cutaneous abscess or pyoderma	27 (44)
Wound infection	4 (7)
Impetigo	3 (5)
Other	1 (2)
Colonization	26 (43)
Site of skin infection (n = 27)	
Head and face	6
Upper extremity	6
Trunk and buttock	7
Lower extremity	8
Other clinical features	
Presence of >1 other medical condition	16 (26)
Previous exposure (<6 mo) to antimicrobial drug	16 (26)
Case-fatality mortality rate	0

*Values are no. (%) of patients unless otherwise indicated. For 2 patients, no microbiologic specimen was available for molecular characterization. See also supplementary material (available from www.genomic.ch/sup6.php). CA-MRSA, community-associated methicillin-resistant *Staphylococcus aureus*.

Strains were rarely resistant to clindamycin (5%), gentamicin (8% only in isolates recovered after 2002), or rifampicin (<1%). Susceptibility to cotrimoxazole was 89% during the first period and 100% during the second period. PVL-positive isolates remained multidrug susceptible throughout the study period, and were distinct from our endemic nosocomial strain.

The Figure, panel** A**, shows the incidence of isolates fulfilling our entry criteria and the proportion of strains producing PVL or harboring SCC*mec* IV or V. An increase in non–multidrug-resistant MRSA was observed during 1994–1997 (incidence 2.3 cases/10,000 admissions in 1997), and a second peak was observed during 2002–2005 (incidence 4.3 cases/10,000 admissions in 2004). Molecular characterization of the 151 strains showed that 124 (82%) harbored either SCC*mec* IV or V. A total of 92 isolates (61%) harbored at least 1 toxin gene, most frequently PVL (n = 60), followed by TSST-1 (n = 22) and exfoliatin A (n = 11). An isolate (ST149-MRSA-IV) from a Libyan patient harbored the PVL and TSST-1 genes (Table 2). A strain with PVL (ST80-MRSA-IV) was isolated in 1994 from a 73-year-old man from Libya. A case of bacteremia with PVL-positive CA-MRSA (ST80-MRSA-IV) was documented in a 28-year-old Tunisian woman who had an abscess of her left forearm in 2000. No case of necrotizing pneumonia was observed.

**Figure:**
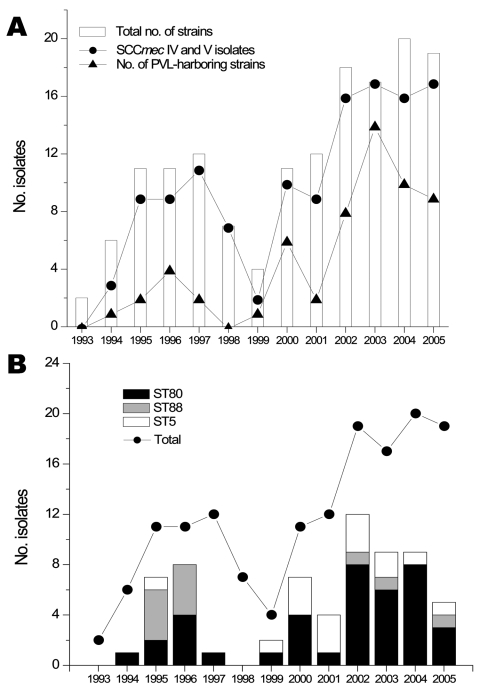
Incidence of non–multidrug-resistant methicillin-resistant *Staphylococcus aureus* (MRSA) strains, Geneva, Switzerland, 1993–2005. A) Number of strains collected since 1993 showing an atypical multidrug-susceptible phenotype (white bars). Also shown are the number of SCC*mec* IV and V (circles) isolates and number of strains containing Panton-Valentine leukocidin (PVL) (triangles). B) Evolution of the 3 most abundant clonotypes (ST80, ST88, and ST5). Despite a constant number of strains isolated since 2002, the proportion of these clones has decreased, which suggests increasing diversity of clones in our population of community acquired–MRSA.

**Table 2:** Molecular characteristics of the non–multidrug-resistant methicillin-resistant *Staphylococcus aureus* isolates, Geneva University Hospitals, 2002–2005*

Isolates	No. strains	PVL	Exfoliatin toxin A	TSST-1	*agr* type				Main MLST types
					1	2	3	4	
SCC*mec* I	21	0	0	11	9	11	1	0	5,149
SCC*mec* II	1	0	0	0	0	1	0	0	ND
SCC*mec* III	1	0	0	0	0	0	1	0	ND
SCC*mec* IV†	109	53	11	9	20	24	65	0	1, 5, 8, 22, 30, 72, 80, 85, 88, 149
SCC*mec* V	15	6	0	1	8‡	3	4	0	1, 30, 59, 85, 152
SCC*mec* NT	4	1	0	1	1	1	2	0	ND
Total§	151	60	11	22	38	40	73	0	

*PVL, Panton-Valentine leukocidin; TSST-1, toxic shock syndrome toxin 1; *agr*, accessory gene regulator; MLST, multilocus sequence typing; SCCmec, staphylococcal cassette chromosome mec; ND, not determined; NT, nontypeable. †Two isolates had recombinases identical to SCC*mec* IVe on the basis of sequence information; 4 isolates were not typeable. ‡One isolate was characterized after PCR amplification and sequencing as previously described (*12*). §A total of 92 strains had toxins, but the total number of toxin genes detected was 93 because 1 isolate contained PVL and TSST-1.

From 1994 through 1999, we identified 14 PVL-positive MRSA isolates. The Figure, panel** B**, shows that CA-MRSA identified during 1993–2002 consisted mainly of 3 clonotypes (ST80, ST88, and ST5). After 2002, these strains were less frequent and the proportion of other clonotypes increased.

The Appendix Figure shows that toxin-harboring strains segregated in 18 multiple-locus variable-number tandem repeat analysis profiles and yielded 14 multilocus sequences types. ST80 (n = 39) was the most abundant type (42% of toxin-producing isolates). Other clusters contained well-described ST5 (TSST-1 or PVL positive), ST30, and ST8 (USA300) strains harboring the PVL gene.

Several epidemiologically linked cases were identified in the second period (Appendix Figure: a cluster of 6 family members with recurrent furunculosis over 5 years (ST80-MRSA-IV), 4 smaller family clusters with the same clone, and 3 family clusters with other clonally related strains (ST5-MRSA-IV, ST59-MRSA-V, ST1-MRSA-V). Two inmates incarcerated in the same cell of the Geneva prison had abscesses caused by the ST8-MRSA-IV (USA300) strain. One outbreak involved 5 neonates and 2 mothers colonized or infected with ST5-MRSA-IV harboring the PVL gene (*5*). We also observed a cluster of 5 patients from Kosovo infected or colonized with a PVL-producing strain (ST152-MRSA-V) resistant to gentamicin and amikacin.

Strains lacking toxins (Appendix Figure, panel** B**) showed a wide diversity of patterns. Most isolates (n = 33) were obtained from the first stain collection and showed many different genetic backgrounds (n = 21).

## Conclusions

We studied 2 collections of non-multiresistant MRSA strains identified over a 13-year period at our institution. Our analysis showed that sporadic PVL-positive CA-MRSA has been isolated in Geneva since 1994; the largest cluster corresponded to ST 80 (SCC*mec* IV, PVL positive); the PVL gene is disseminated in many genetic backgrounds; strains showed diversity of genomic content; several epidemiologic clusters were identified; and many cases were linked to migration and travel.

Our data showed that resistance to fusidic acid or susceptibility to gentamicin should not be used as phenotypic criteria for CA-MRSA in Europe. For example, gentamicin-resistant ST152-MRSA-V found in 5 patients from Kosovo is common.

The number of genetic profiles identified appears particularly high, which is similar to profiles described in European countries, northern Africa, Oceania, and the Americas. In contrast to CA-MRSA epidemiology described as homogeneous in Australia (*13*), some parts of the United States (*14*), or Sweden (*15*), our local epidemiology appears more heterogeneous.

Frequently observed importation of CA-MRSA may enhance genetic exchange between strains and supports the need for active surveillance. Most CA-MRSA remained susceptible to many antimicrobial drugs, but genetic exchange between strains resulting in acquisition of resistance determinants in CA-MRSA or transfer of virulence markers into HA-MRSA are important concerns.

Our study has limitations. First, we used 2 strain collections. The collection obtained before 2003 may have omitted gentamicin-resistant CA-MRSA strains (e.g., ST152). Second, retrospective case ascertainment does not distinguish invasive from colonizing strains in all patients. Finally, we cannot exclude detection bias caused by our active MRSA screening policy (*6**,**7*).

In summary, increasing incidence of PVL-producing type IV CA-MRSA isolates is worrisome and indicates emergence of new MRSA lineages with a particular fitness for community transmission. Further epidemiologic and molecular typing studies are needed to document CA-MRSA carriage and infection rates and implement adequate infection control guidelines.

## Supplementary Material

Appendix Figure Clustering trees. A) Trees obtained by using multiple-locus variable-number tandem repeat analysis (MLVA)–based genotyping for strains of Staphylococcus aureus harboring clinically important toxins, Geneva, Switzerland, 1993–2005. Year of isolation, staphylococcal cassette chromosome mec (SCCmec) type, toxin content, multiple locus sequence type, and accessory gene regulator (agr) types are also indicated for each strain (year/SCCmec/ST/agr). Major clusters appear in gray. ND, not determined; NT, nontypeable. *Clonal strains isolated from the familial cluster of ST80-MRSA-IV harboring the Panton-Valentine leukocidin (PVL) gene. **First ST80-MRSA-IV harboring the PVL gene isolated in 1994. ***Aypical ST149 strain clustering with other ST149 isolates, showing 2 toxins. ^A^ single locus variant of ST395; ^B^ small familial clusters of clonal strains isolated in 2 pairs of relatives; ^C^ and ^D^ 2 pairs of clonal strains from a neonatology cluster previously described (*7*); ^E^ patients returning from New York infected with USA300; ^F^ clusters of isolates from 2 prison inmates; ^G^ the only patient (intravenous drug user) with a strain highly related to USA400; ^H^ isolate showing molecular content of the ST59 Pacific clone. Scale bar (lower left) shows relative distance between strains. B). Trees obtained by using MLVA-based genotyping for strains devoid of clinically important toxins. Year of isolation, SCCmec type, toxin content, MLST, and agr types are also indicated for each strain (year/SCCmec/ST/agr). ^A^ familial cluster of ST1-MRSA-V composed of a mother and her 2 children; ^B^ control strain MW2 (USA400); ^C^ control strain ST228-MRSA-I representing the common nosocomial strain in our area. Scale bar (lower left) shows relative distance between strains.
