# Supplementary figures and images for: Methicillin-Resistant Staphylococcus aureus, Geneva, Switzerland, 1993–2005
Source: Emerg Infect Dis. 2008 Feb;14(2):304–7. doi: 10.3201/eid1402.070229 (PMC2600191; doi:10.3201/eid1402.070229)

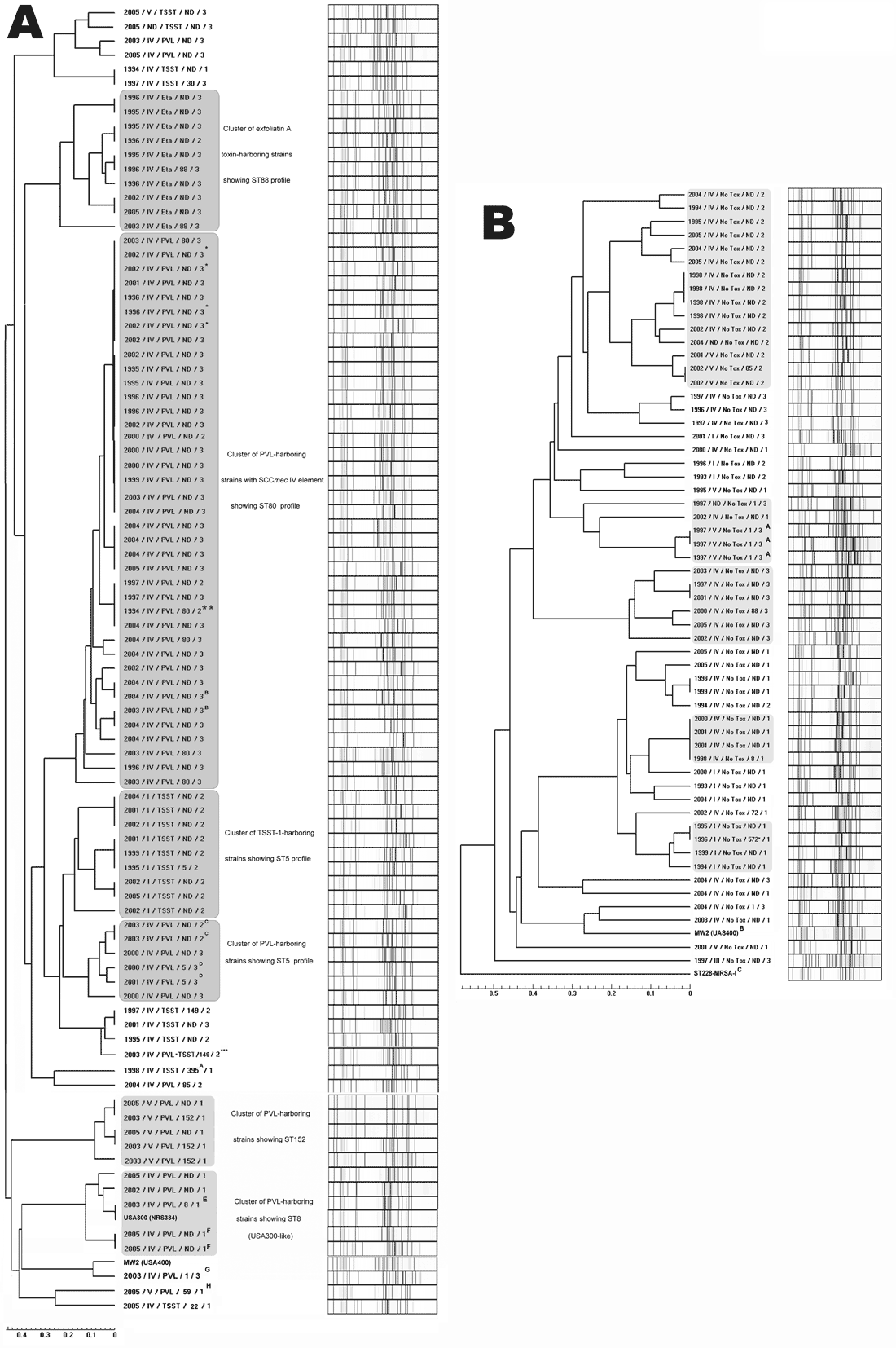

Supplement: Appendix Figure — Clustering trees. A) Trees obtained by using multiple-locus variable-number tandem repeat analysis (MLVA)–based genotyping for strains of Staphylococcus aureus harboring clinically important toxins, Geneva, Switzerland, 1993–2005. Year of isolation, staphylococcal cassette chromosome mec (SCCmec) type, toxin content, multiple locus sequence type, and accessory gene regulator (agr) types are also indicated for each strain (year/SCCmec/ST/agr). Major clusters appear in gray. ND, not determined; NT, nontypeable. *Clonal strains isolated from the familial cluster of ST80-MRSA-IV harboring the Panton-Valentine leukocidin (PVL) gene. **First ST80-MRSA-IV harboring the PVL gene isolated in 1994. ***Aypical ST149 strain clustering with other ST149 isolates, showing 2 toxins. A single locus variant of ST395; B small familial clusters of clonal strains isolated in 2 pairs of relatives; C and D 2 pairs of clonal strains from a neonatology cluster previously described (7); E patients returning from New York infected with USA300; F clusters of isolates from 2 prison inmates; G the only patient (intravenous drug user) with a strain highly related to USA400; H isolate showing molecular content of the ST59 Pacific clone. Scale bar (lower left) shows relative distance between strains. B). Trees obtained by using MLVA-based genotyping for strains devoid of clinically important toxins. Year of isolation, SCCmec type, toxin content, MLST, and agr types are also indicated for each strain (year/SCCmec/ST/agr). A familial cluster of ST1-MRSA-V composed of a mother and her 2 children; B control strain MW2 (USA400); C control strain ST228-MRSA-I representing the common nosocomial strain in our area. Scale bar (lower left) shows relative distance between strains. [file 07-0229_app-s1.gif]
